# Dissecting the Genomic Architecture of Resistance to *Eimeria maxima* Parasitism in the Chicken

**DOI:** 10.3389/fgene.2018.00528

**Published:** 2018-11-26

**Authors:** Kay Boulton, Matthew J. Nolan, Zhiguang Wu, Valentina Riggio, Oswald Matika, Kimberley Harman, Paul M. Hocking, Nat Bumstead, Pat Hesketh, Andrew Archer, Stephen C. Bishop, Pete Kaiser, Fiona M. Tomley, David A. Hume, Adrian L. Smith, Damer P. Blake, Androniki Psifidi

**Affiliations:** ^1^The Roslin Institute and Royal (Dick) School of Veterinary Studies, The University of Edinburgh, Edinburgh, United Kingdom; ^2^Department of Pathobiology and Population Sciences, Royal Veterinary College, University of London, London, United Kingdom; ^3^Enteric Immunology Group and Genetics and Genomics Group, Pirbright Institute, Woking, United Kingdom; ^4^Mater Research Institute, The University of Queensland, Brisbane, St. Lucia, QLD, Australia; ^5^Department of Zoology, Sir Peter Medawar Building for Pathogen Research, University of Oxford, Oxford, United Kingdom; ^6^Department of Clinical Sciences and Services, Royal Veterinary College, University of London, Hatfield, United Kingdom

**Keywords:** intercross, backcross, *Eimeria maxima*, QTL, resistance, interleukin-10, oocyst output

## Abstract

Coccidiosis in poultry, caused by protozoan parasites of the genus *Eimeria*, is an intestinal disease with substantial economic impact. With the use of anticoccidial drugs under public and political pressure, and the comparatively higher cost of live-attenuated vaccines, an attractive complementary strategy for control is to breed chickens with increased resistance to *Eimeria* parasitism. Prior infection with *Eimeria maxima* leads to complete immunity against challenge with homologous strains, but only partial resistance to challenge with antigenically diverse heterologous strains. We investigate the genetic architecture of avian resistance to *E. maxima* primary infection and heterologous strain secondary challenge using White Leghorn populations of derived inbred lines, C.B12 and 15I, known to differ in susceptibility to the parasite. An intercross population was infected with *E. maxima* Houghton (H) strain, followed 3 weeks later by *E. maxima* Weybridge (W) strain challenge, while a backcross population received a single *E. maxima* W infection. The phenotypes measured were parasite replication (counting fecal oocyst output or qPCR for parasite numbers in intestinal tissue), intestinal lesion score (gross pathology, scale 0–4), and for the backcross only, serum interleukin-10 (IL-10) levels. Birds were genotyped using a high density genome-wide DNA array (600K, Affymetrix). Genome-wide association study located associations on chromosomes 1, 2, 3, and 5 following primary infection in the backcross population, and a suggestive association on chromosome 1 following heterologous *E. maxima* W challenge in the intercross population. This mapped several megabases away from the quantitative trait locus (QTL) linked to the backcross primary W strain infection, suggesting different underlying mechanisms for the primary- and heterologous secondary- responses. Underlying pathways for those genes located in the respective QTL for resistance to primary infection and protection against heterologous challenge were related mainly to immune response, with IL-10 signaling in the backcross primary infection being the most significant. Additionally, the identified markers associated with IL-10 levels exhibited significant additive genetic variance. We suggest this is a phenotype of interest to the outcome of challenge, being scalable in live birds and negating the requirement for single-bird cages, fecal oocyst counts, or slaughter for sampling (qPCR).

## Introduction

Coccidiosis is an intestinal disease caused by intracellular protozoan parasites of the genus *Eimeria* (Shirley et al., 2005). The control of coccidiosis is a challenge to the international poultry industry, with economic losses estimated at USD 3 billion annually (Dalloul and Lillehoj, 2006). Current control of coccidiosis relies on the prophylactic use of anticoccidial drugs, or vaccination with formulations of live wild-type or attenuated parasites (Crouch et al., 2003; McDonald and Shirley, 2009). However, use of some anticoccidial drugs has been curtailed by legislation, while the limited production capacity and costs of live attenuated vaccines compromise their utility in broiler flocks (Hong et al., 2006). Thus, there is a need for complementary strategies to control coccidiosis in poultry. A promising approach would be to breed chickens for increased genetic resistance and increased vaccine response to *Eimeria* parasitism since there is evidence for relevant host genetic variation (Johnson et al., 1986; Bumstead and Millard, 1992).

Coccidiosis in poultry is caused by seven distinct *Eimeria* species (Reid et al., 2014), with *Eimeria maxima* being one of the most common causes of coccidiosis in commercial broilers. Immunity introduced by primary infection (vaccination) against *E. maxima* is commonly strain-specific, with immune escape contributing to sub-clinical coccidiosis symptoms that include decreased feed conversion efficiency, marked weight loss and low performance (Fitz-Coy, 1992; Blake et al., 2005).

Johnson et al. (1986) demonstrated variance in coccidiosis susceptibility in chickens as a prerequisite to selective breeding for resistance. A subsequent study using several inbred White Leghorn lines established variance for benchmark phenotypes when chickens were infected with controlled doses of *Eimeria* spp. (Bumstead and Millard, 1987, Bumstead and Millard, 1992). The between-line variation observed in oocyst production by the different lines was not correlated with weight loss or mortality, indicating that within-trait observations were a result of effect accommodation rather than parasite restriction. The greatest differences in parasite replication (PR) were between lines 15I and C major histocompatibility complex (MHC) haplotype B12 (C.B12) chickens that produced relatively high and low numbers of oocysts, respectively (Bumstead and Millard, 1987; Smith et al., 2002). Most notably, primary infection with the Houghton or Weybridge reference *E. maxima* strains induce 100% protection against secondary homologous challenge in 15I and C line chickens (Smith et al., 2002). However, the outcome of heterologous challenge varied by parasite strain and host genotype combination (Smith et al., 2002; Blake et al., 2004, 2005). Regardless of the substantial financial losses to industry caused by coccidiosis, few studies have attempted to identify quantitative trait loci (QTL) for resistance to *E. maxima* infection and there are no relevant studies on the genetics of heterologous secondary challenge response.

The present study extends previous work in inbred chicken lines to determine the genetic architecture of *E. maxima* resistance, i.e., lack of PR, and protection against secondary challenge with a heterologous *E. maxima* strain. First, an F2 intercross of inbred White Leghorn chicken lines C.B12 × 15I were initially infected with *E. maxima* H, followed 3 weeks later by challenge with *E. maxima* W to investigate response to challenge with the heterologous strain. Fecal oocyst output was counted to determine severity of challenge. Second, a backcross population from the same two inbred lines [(C.B12 × 15I) × C.B12] was infected with *E. maxima* W to study primary resistance to parasitism. Three phenotypes were determined for these birds following infection: PR by qPCR for parasite numbers in intestinal tissue, intestinal lesion score (LS) (gross pathology, scale 0–4) and levels of serum interleukin-10 (IL-10), a novel biomarker, found to be positively correlated with the pathology trait in chickens infected with E. *tenella* (Wu et al., 2016; Boulton et al., 2018). All birds were then genotyped using a 600K Affymetrix^®^ Axiom^®^ HD array (Kranis et al., 2013), enabling genome-wide association studies (GWASs), followed by pathway analysis to identify candidate genomic regions, pathways, networks and genes for resistance to *E. maxima* primary infection and effective responses to challenge with a heterologous strain.

## Materials and Methods

### Ethics Statement

These trials were conducted under Home Office Project Licence in accordance with Home Office regulations under the Animals (Scientific Procedures) Act 1986 and the guidelines set down by the Institute for Animal Health and RVC Animal Welfare and Ethical Review Bodies.

### Parasites

The *E. maxima* Houghton (H) and Weybridge (W) strains were used throughout these studies (Norton and Hein, 1976). Routine parasite passage, sporulation, and dose preparation were undertaken as described previously (Eckert et al., 1995) using specific pathogen free Light Sussex or Lohman LSL chickens. Oocysts were used within 1 month of harvest.

### Animals

Inbred chicken lines 15I and C derived from White Leghorn flocks at USDA-ARS Avian Disease and Oncology Laboratory in East Lansing, MI, United States, were maintained by random mating within the specified-pathogen-free (SPF) flocks at the Pirbright Institute [formerly the Institute for Animal Health (IAH)], United Kingdom since 1962 and 1969, respectively.

F2 intercross birds (*n* = 195) were generated by crossing nine F1 (C.B12 × 15I) male progeny with 27 unrelated F1 female progeny at the IAH (Compton site). Six birds from each of the two parental lines, 15I and C.B12, were also hatched and kept under the same experimental conditions as F2 (individual cages post-challenge).

To generate the backcross (*n* = 214), 20 F1 (C.B12 × 15I) male progeny were crossed with 100 unrelated C.B12 line females. The breeding was performed in the SPF Bumstead facility at the Roslin Institute, The University of Edinburgh, United Kingdom. Day old chicks were transported in isolated SPF containment to the Royal Veterinary College poultry barn, University of London, United Kingdom, where the primary infection with *E. maxima* W sporulated oocysts were conducted in floor pens.

### *Eimeria maxima* Challenge Experiments

#### Intercross Population

F2 intercross (*n* = 195), and 12 parental line birds were initially infected by oral gavage with 100 sporulated oocysts of *E. maxima* H at 25 days of age and moved to individual cages. Feces were collected from each bird on a daily basis during the 5–10 days post-challenge (pi) period following infection. Three weeks later (47 days of age) a secondary challenge was initiated by oral gavage of 250 sporulated oocysts of *E. maxima* W. Feces were again collected from each bird on a daily basis during the 5–10 day post-challenge period.

#### Backcross Population

At 21 days of age, chickens were inoculated by oral gavage with either 1 ml distilled water (control group, *n* = 20) or 100 sporulated oocysts of *E. maxima* W (infected group, *n* = 194). To avoid cross-infection the control group was housed separately. Birds were euthanised humanely at day 7 pi, coinciding with the peak pathological effects of *E. maxima* (Rothwell et al., 2004), providing the greatest sensitivity for parasite genome detection (Blake et al., 2006). A blood sample from each bird was collected post-mortem *via* aortic rupture into 1.5 ml Sigma-Aldrich (Dorset, United Kingdom) microcentrifuge tubes. Bijou tubes (7 ml Sterilin^TM^) containing 5–10 volumes of room temperature RNA*later*^®^ (Life Technologies, Carlsbad, CA, United States) were used to store 5.0 cm of intestinal tissue and content from either side of Meckel’s diverticulum.

### Phenotyping

Individual oocyst output was used to study the outcome of the *E. maxima* H primary infection and secondary heterologous *E. maxima* W challenge in the intercross chicken population. Oocysts were quantified daily (5 to 10 days post- infection and challenge) using a microscope and saturated salt flotation in a McMaster counting chamber (Eckert et al., 1995; Smith et al., 2002). Daily totals were combined to provide a total count for oocyst output per bird for both the primary infection and secondary challenge. Oocyst counts were log-transformed to approximate normal distribution.

The phenotypes used to study resistance to *E. maxima* W primary infection in the backcross population were relative intestinal *Eimeria* genome copy number (PR, measured using quantitative PCR as parasite genomes per host chicken genome), intestinal LS (pathology, on a scale 0–4), and serum IL-10 level (IL-10). Quantitative real-time PCR targeting the *E. maxima* microneme protein 1 (*EmMIC1*) and *Gallus gallus* β-actin (*actb*) loci was performed using total genomic DNA extracted from a 10 cm length of intestinal tissue centered on Meckel’s diverticulum using a DNeasy Blood and Tissue kit (Qiagen, Hilden, Germany). Briefly, each complete tissue sample was disaggregated using a Qiagen TissueRuptor and an aliquot was processed for extraction of combined host and parasite DNA (see Blake et al., 2006, for full details). A CFX96 Touch^®^ Real-Time PCR Detection System (Bio-Rad Laboratories, Hercules, CA, United States), was used to amplify each sample in triplicate (Nolan et al., 2015), with an additional Bead-Beater homogenization step prior to buffer ATL treatment (including 1 volume 0.4–0.6 mm glass beads, 3,000 oscillations per minute for 1 min). Intestinal pathology was assessed by the same experienced operator scoring lesions according to Johnson and Reid (1970). A capture ELISA was used to measure IL-10, employing ROS-AV164 and biotinylated ROS-AV163 as capture and detection antibodies, respectively (see Wu et al., 2016, for full details). IL-10 levels and parasite genome numbers were log-transformed to approximate normal distribution.

### Phenotypic Correlations

Following log-transformation for PR and IL-10, all backcross phenotypic traits were rescaled to modify the unit of measurement differences. Then, fitting host sex as a fixed effect in a multivariate linear model, phenotypic correlations (r_P_) were estimated using ASReml 4.1 (Gilmour et al., 2015).

### Genome-Wide Association Studies

Sixty-seven F2 birds exhibiting the most extreme phenotypes, plus the 12 intercross parental line birds and the entire backcross generation were genotyped using the 600K Affymetrix^®^ Axiom^®^ HD genotyping array (Kranis et al., 2013). Although each data set was analyzed separately, the same GWAS steps were used for both populations. The marker genotype data were subjected to quality control measures using the thresholds: minor allele frequency < 0.02 and call rate > 90%. Deviation from Hardy–Weinberg equilibrium was not considered a reason for excluding markers since these were experimental populations of inbred lines. After quality control 203,845 intercross and 204,072 backcross markers remained and were used, respectively, to generate separate intercross and backcross genomic relationship matrixes (GRMs) to investigate the presence of population stratification. Next, each GRM was converted to a distance matrix that was analyzed with a classical multidimensional scaling using the GenABEL package of R (Aulchenko et al., 2007) to obtain principal components. These analyses revealed three principal components in the intercross population (one for each parental line and one for F2 birds), but no substructure in the backcross. GWAS for each trait were then conducted using GenABEL based on a mixed model, with the population principal components fitted as a co-variate (intercross population only), sex fitted as a fixed effect in both studies, and GRM fitted as a random polygenic effect to adjust for population sub-structure. In the case of GWAS for heterologous secondary challenge response, the oocyst output following the first challenge was also fitted as a covariate to account for the effect of the first challenge. After Bonferroni correction for multiple testing, significance thresholds were *P* ≤ 2.45 × 10^-7^ and *P* ≤ 4.90 × 10^-6^ for genome-wide (*P* ≤ 0.05) and suggestive (namely one false positive per genome scan) significant levels, respectively corresponding to -log_10_ (*P*) of 6.61 and 5.30. The extent of linkage disequilibrium (LD) between significant markers located on the same chromosome regions was calculated using the r-square statistic of PLINK v1.09 (Purcell et al., 2007).

Effects of the significant markers identified in each GWAS were re-estimated in ASReml 4.1 (Gilmour et al., 2015) by individually fitting the markers as fixed effects in the same model as used for GWAS analyses. Effects were calculated as follows: additive effect, a = (AA – BB)/2; dominance effect, d = AB-((AA + BB)/2), where AA, BB, and AB were the predicted trait values for each genotype class.

All significant markers identified in GWAS for responses to primary infection and secondary *E. maxima* W challenge were mapped to the reference *Gallus gallus domesticus* genome and annotated using the variant effect predictor^1^ tool within the Ensembl (genome browser 92) database and the Gal-gal5 assembly^2^. Furthermore, genes located within 100 kb up- and down-stream of the significant markers were annotated using the BioMart data mining tool^3^ and the Gal-gal5 assembly. This method of annotation enabled all genes located in the vicinity of the identified significant markers to be identified and cataloged.

### Re-sequencing Data Analysis

To identify possible protein-coding genes associated with the detected QTL, genomic sequences in the regions of interest from the line 15I and C.B12 chickens were compared. The two parental chicken lines were entirely re-sequenced at 15–20 fold coverage, using pools of 10 individuals per line, performed on an Illumina GAIIx platform using a paired-end protocol (Krämer et al., 2014). Re-sequencing data of the candidate regions (i.e., 1 kb up- and downstream of the candidate gene end sites), for resistance to primary infection and heterologous challenge derived from intercross and backcross analyses, were then extracted and examined separately. Using the Mpileup tool for marker calling (SAMtools v0.1.7; Li et al., 2009), single nucleotide variants (SNVs) between the two parental lines and the reference genome in these regions were detected. These were then annotated using the same variant effect predictor software as above. Information for all SNV [intergenic, intronic, exonic, splicing, 3′ and 5 untranslated regions (3′ UTR, 5′ UTR)] present in the regions of interest were collated. Intergenic, intronic, and exonic synonymous variants were then filtered out along with SNV that were common in the two parental lines but different from the reference genome. Thus, only sites that were different between the parental lines and had an effect on the coding sequence (nonsense, missense, splicing) or a potential effect on the gene expression (3′ UTR and 5′ UTR) were retained for further study.

### Pathway, Network, and Functional Enrichment Analyses

Identification of potential canonical pathways and networks underlying the candidate genomic regions associated with outcomes of primary infection and heterologous secondary *E. maxima* challenge were performed using the ingenuity pathway analysis (IPA) program^4^. IPA constructs multiple possible upstream regulators, pathways, and networks that serve as hypotheses for the biological mechanism underlying the phenotypes based on a large-scale causal network derived from the Ingenuity Knowledge Base. After correcting for a baseline threshold and calculating statistical significance, the most likely pathways involved are inferred (Krämer et al., 2014). The constructed networks can then be ranked using their IPA score based on the *P*-values obtained using Fisher’s exact test [IPA score or *P*-score = -log_10_ (*P*-value)].

The gene lists for each phenotype were also analyzed using the Database for Annotation, Visualization and Integrated Discovery (DAVID; Dennis et al., 2003). To understand the biological meaning behind these genes, gene ontology (GO) was determined, and functional annotation clustering analysis was performed using the integral *G. gallus* background. The enrichment score (ES) of DAVID is a modified Fisher exact *P*-value calculated by the software, with higher ES reflecting more enriched clusters. An ES > 1 means that the functional category is overrepresented.

## Results

### Descriptive Statistics

**FIGURE 1:**
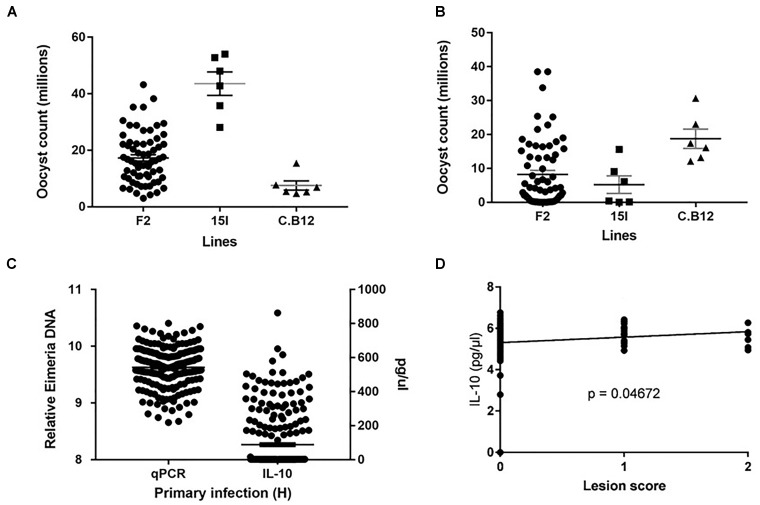
Distribution of the intercross *Eimeria maxima* replication in **(A)** primary infection with Houghton and **(B)** secondary challenge with Weybridge heterologous strain for F2 and parental lines 14I and C.B12. **(C)** Backcross relative DNA and IL-10 distributions. **(D)** Backcross Lesion Score (LS) and (log_10_) IL-10 correlations, with significance value (*P*). For (**A–C**), black horizontal bars represent the distribution means and gray error bars the SE of the mean.

Phenotypic distributions for oocyst counts following primary infection with *E. maxima* H and secondary challenge with *E. maxima* W in the intercross and parental populations along with relative DNA and IL-10 levels in the backcross populations after primary infection with *E. maxima* W are presented in Figures 1A–C. After primary infection the pure line C.B12 birds produced fewer *E. maxima* oocyst counts compared to the pure line 15I and F2 birds, with the highest oocyst output recorded in the pure line 15I group. Conversely, inverse findings regarding oocyst output were recorded in the two parental lines following heterologous secondary strain challenge. These results agree with previous findings that show line C.B12 birds develop no cross protection between primary H and secondary W strain challenges, while line 15I birds develop significant cross-protection when infected in this order (Smith et al., 2002; Blake et al., 2005). As expected, for both primary and secondary challenges F2 intercross population oocyst count level was intermediate between those of the two parental lines.

Among the backcross chickens, following infection with *E. maxima* W, phenotypic scores for intestinal lesions were low (0–2), however significant variance (*P* = 0.05) was noted (Table 1). Estimated phenotypic correlations between the three measured traits ranged from 0.8 to 0.15, with only the correlation between LS and IL-10 being statistically significant (r_LS,IL-10_ = 0.15 ± 0.07; Figure 1D and Table 1).

**Table 1:** Genetic covariance/variance/correlation matrix (± SE), for the backcross primary *E. maxima* infection trial.

	PR	LS	IL-10
PR	*0.92 (0.09)*	0.12 (0.07)	0.08 (0.07)
LS	0.11 (0.07)	*0.90 (0.09)*	*0.15 (0.07)*
IL-10	0.07 (0.07)	*0.14 (0.07)*	*1.0 (0.10)*

*Significant values are highlighted in italics. Covariances are presented below the diagonal, variances are shaded on the diagonal, with between-trait correlations above. Measured traits are parasite replication per host genome (PR), lesion score (LS), and serum interleukin-10 (IL-10)*.

### Genome-Wide Association Studies

#### Intercross Study

**FIGURE 2:**
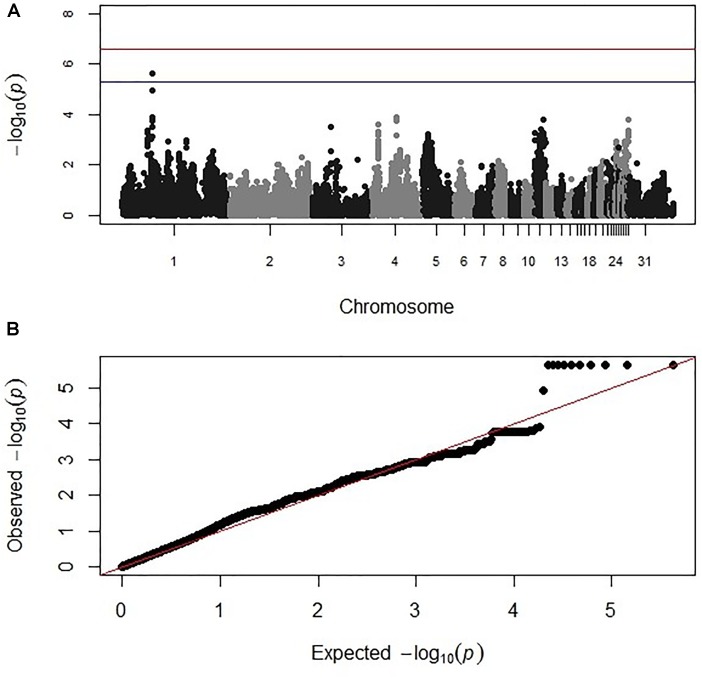
**(A)** Manhattan and **(B)** corresponding Q–Q plot for GWAS for oocyst output measured from the intercross chickens following heterologous secondary challenge. The –log_10_ *P*-value (on the y axis) indicating genome-wide significance is represented by the red line, while the blue line represents suggestive significance. The positions of the markers analyzed for the 28 main chicken autosomes (1–28) plus the sex chromosomes Z and W (29 and 30 respectively) and microchromosomes (31), are represented on the x axis. In **(B)**, the expected chi-squared (χ^2^) values are plotted on the x axis, whereas the observed χ^2^ values are presented on the y axis, with the red line indicating the anticipated slope.

Genome-wide association study analysis for oocyst output following primary infection of the intercross population with *E. maxima* H did not reveal significant associations after the strict Bonferroni correction. However, an association with markers on chromosome 2, just below the suggestive threshold was reported (results not shown). GWAS analysis following secondary challenge with the heterologous *E. maxima* W strain identified 11 markers on chromosome 1, all having suggestive associations with the trait in the intercross population. These 11 markers belonged to the same LD block (499 bp, r^2^ = 1; Figure 2 and Table 2). The corresponding Q–Q plot for the GWAS intercross result is found in Figure 2.

**Table 2:** Details of GWAS-identified and animal model-verified significant markers for oocyst output (OoC) from the intercross chickens following secondary challenge with the heterologous *E. maxima* W strain.

Trait	Marker	Location (Chr:mb)	G_A_ (*P*-value)
OoC	Affx-50382738	1:55145901	0.82 (<0.001)
	Affx-50382750	1:55150313	0.82 (<0.001)
	Affx-50382770	1:55160683	0.82 (<0.001)
	Affx-50382803	1:55177556	0.82 (<0.001)
	Affx-50382825	1:55192552	0.82 (<0.001)
	Affx-50382867	1:55217464	0.82 (<0.001)
	Affx-50382871	1:55220484	0.82 (<0.001)
	Affx-50382878	1:55224572	0.82 (<0.001)
	Affx-50382880	1:55225227	0.82 (<0.001)
	Affx-50382881	1:55225894	0.82 (<0.001)
	Affx-50382786	1:55169757	0.82 (<0.001)

*Details provided: Affymetrix marker identifier; chromosome and position of markers in the Galgal5 assembly (Chr:mb); additive genetic effects (G_*A*_), with significance values (*P*-value)*.

The 11 significant markers associated with the outcome of secondary challenge by the heterologous *E. maxima* strain were all located in intronic, upstream, and downstream regions of the *phenylalanine hydroxylase* (*PAH*) gene (Supplementary Table S1). In the 0.5 Mb candidate region for enhanced response to heterologous secondary *E. maxima* challenge only 16 protein coding genes were located (Supplementary Table S2).

#### Backcross Study

**FIGURE 3:**
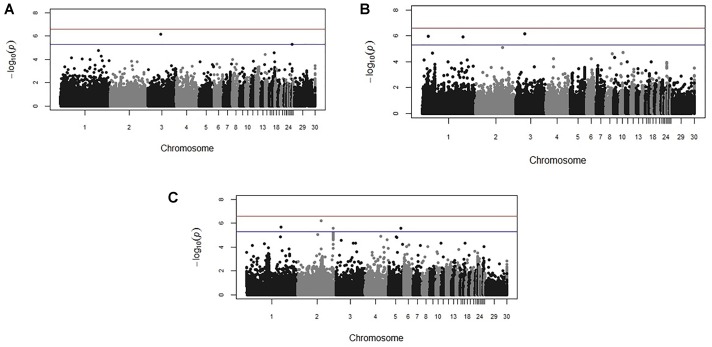
Manhattan plots from the backcross chicken response to *E. maxima* infection GWAS for the three measured traits: **(A)** parasite replication (PR), **(B)** LS, and **(C)** IL-10. The –log_10_ *P*-value (vertical axis) indicating genome-wide significance is represented by the red line, while the blue line represents genome-wide suggestive significance. The positions of the markers analyzed for the 28 main chicken autosomes (1–28) plus the sex chromosomes Z and W (29 and 30 respectively) and microchromosomes (31), are represented on the horizontal axes.

**FIGURE 4:**
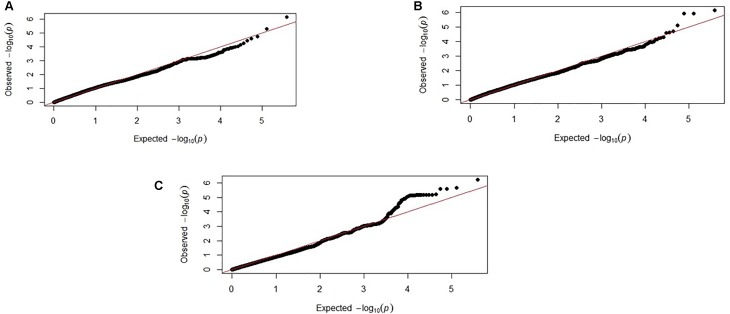
Corresponding Q–Q plots from the backcross chicken response to *E. maxima* infection GWAS of backcross chickens for the three measured traits: **(A)** PR, **(B)** LS, and **(C)** IL-10. The expected chi-squared (χ^2^) values are plotted on the x-axis. The observed χ^2^ values are presented on the y-axis, with the red line indicating the anticipated slope.

Genome-wide association study results for resistance to *E. maxima* W primary infection in the backcross population revealed several of significant genomic associations for each of the measured phenotypes. However, there was no overlap of the candidate genomic regions linked to parasite reproduction, intestinal pathology, or IL-10 induction (Figure 3 and Table 3). Specifically, a single marker on chromosome 3 had a suggestive association with PR (Figure 3A and Table 3). Four suggestive marker associations were identified with markers on chromosomes 1, 2, and 3 for intestinal pathology (i.e., lesion damage; Figure 3B and Table 3). A further four associations were found for IL-10 on chromosomes 1, 2, and 5 (Figure 3C and Table 3). None of the markers found on chromosome 2 for LS and IL-10 were in common, nor were they in LD. However, the candidate QTL region for IL-10 on chromosome 2 was in proximity with an intercross marker found following primary infection with *E. maxima* H in the intercross population that falls below the suggestive threshold. The corresponding Q–Q plots for GWAS are displayed in Figure 4. All significant markers identified in both studies exhibited significant (*P* < 0.01) additive genetic effects (Table 3).

**Table 3:** Details of GWAS-identified and animal model-verified significant markers from the backcross chicken response to *E. maxima* primary infection.

Trait	Marker	Location Chr:mb	G_A_ (*P*-value)
PR	Affx-51275363	3:49284591	-0.66 (<0.001)
LS	Affx-51243371	3:31960902	0.67 (<0.001)
	Affx-50321421	1:20945328	0.73 (<0.001)
	Affx-50226161	1:150098597	0.57 (<0.001)
	Affx-51010702	2:98848476	0.54 (<0.001)
IL-10	Affx-50999702	2:92008902	-1.49 (<0.001)
	Affx-50194384	1:132582437	-1.36 (<0.001)
	Affx-51587399	5:47737604	-1.76 (<0.001)
	Affx-50857122	2:136484786	1.42 (0.002)

*Measured traits – parasite replication per host genome (PR), Lesion Score (LS), and serum interleukin-10 (IL-10). Details provided: Affymetrix marker identifier; chromosome and position of markers in the Gal-gal5 assembly (Chr:mb); the additive genetic effect (G_A_) and significance values (*P*-value)*.

All of the significant markers identified for resistance to primary *E. maxima* W infection in the backcross population were located in intronic or intergenic regions (Supplementary Table S3). The candidate regions for response to primary *E. maxima* W infection contains a small number of genes: 36 protein-coding genes and four microRNAs (Supplementary Table S4).

### Resequencing Analysis

In total, 3,230 variants were identified in the candidate regions associated with resistance to primary *E. maxima* infections. SNV located in exonic regions accounted for less than 3% of the total, while the remaining SNV (97%) were located in intronic, upstream, and downstream regions. Genes with SNVs that could potentially lead to non-functional transcripts were not detected. However, six genes contained missense SNVs that may affect the function of the encoded proteins. More specifically, *LONRF2, CHST10, PDCL3*, and *TBC1D8* genes on chromosome 1, *FAM69C* on chromosome 2, and *IPCEF1* on chromosome 3 had missense with moderate effect SNVs. Also, these genes contained 3′/5′ UTR variants that may affect the expression of these genes. Details of the missense variants identified in the candidate regions for *E. maxima* resistance to primary infection are presented in Supplementary Table S5.

In total, 2,165 SNV were detected in the candidate region on chromosome 1 for the response to heterologous secondary *E. maxima* W challenge. Most of the identified SNV (95%) were located in intronic, upstream and downstream regions; 5% were located in exonic regions, mostly in 3′ and 5′ UTR regions. Nevertheless, three genes (*PMCH, TBXAS1, THL3*) containing missense variants with moderate effects as well as 3′/5′UTR variants were detected. Details of the missense variants identified in the candidate regions for heterologous secondary *E. maxima* W challenge are presented in Supplementary Table S6.

### Pathway, Network, and Functional Enrichment Analyses

**FIGURE 5:**
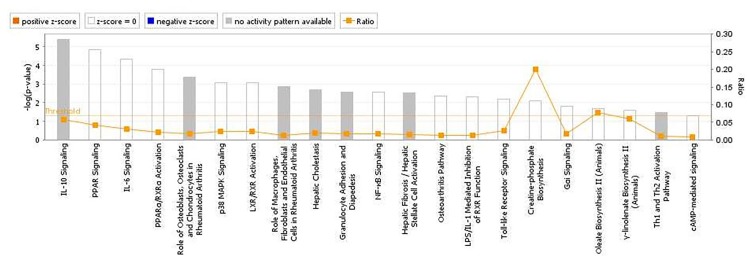
Canonical pathways determined from ingenuity pathway analysis (IPA) of the candidate markers identified in the backcross chicken GWAS for *E. maxima* resistance to primary challenge.

**FIGURE 6:**
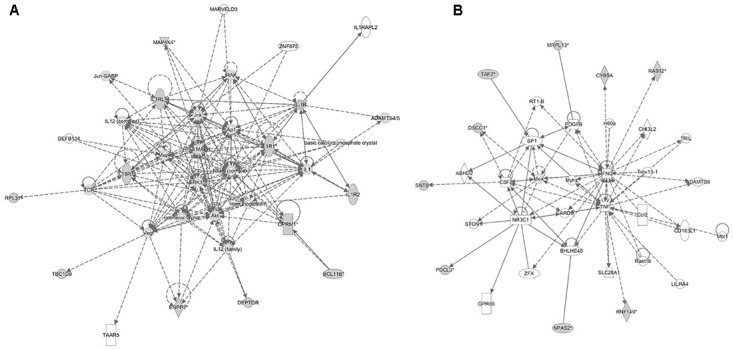
Molecular interaction networks constructed from the canonical pathways identified in the backcross infection response relate to **(A)** inflammatory response and disease and **(B)** cell death and survival, cellular compromise and cell cycle.

The analyses for resistance to primary *E. maxima* infection revealed pathway enrichment for immune response involvement, including IL-10, interleukin-6 (IL-6), nuclear factor kappa-light-chain-enhancer of activated B cells (NF-κb) and toll like receptor signaling (Figure 5). Using the list of candidate region genes, two networks were constructed, comprising molecular interactions related to inflammatory response and disease, cell death and survival, cellular compromise, and cell cycle (IPA scores = 25; Figures 6A,B). A single enriched cluster was found, related to immune response linked to interleukin-1 (IL-1), Toll/IL1 response and cytokine-cytokine receptor response (ES = 2.2, with *IL1R1, IL1RL1, IL2R, IL19R18, PTPRM*, and *COL14A* genes involved).

**FIGURE 7:**
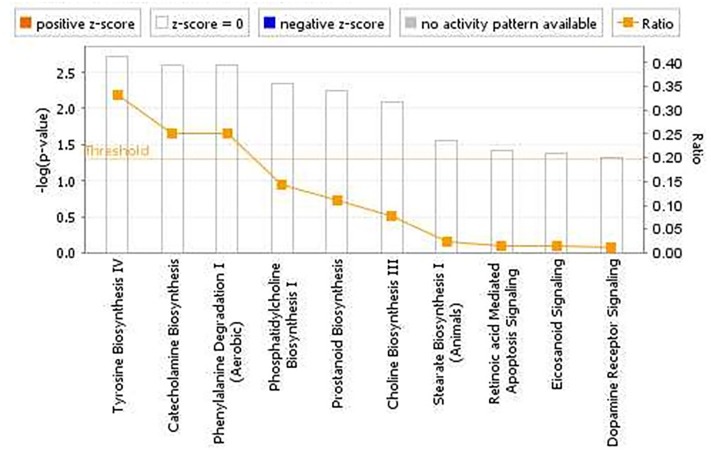
Canonical pathways determined from IPA of the candidate markers identified in the intercross chickens GWAS for *E. maxima* resistance to heterologous secondary challenge.

**FIGURE 8:**
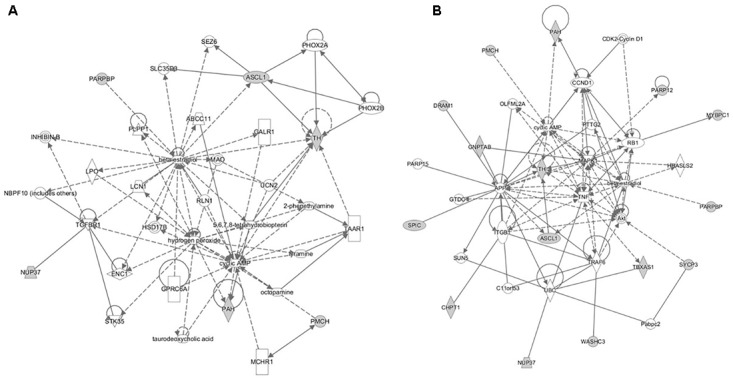
The gene networks subsequently constructed from the intercross heterologous secondary challenge relate to **(A)** cell signaling, nucleic acid metabolism and small molecule biochemistry and **(B)** cellular development, tissue development, and function.

The pathway analyses for response to heterologous *E. maxima* W strain secondary challenge revealed enrichment for both immune (prostanoid biosynthesis, retinoic acid mediated apoptosis signaling, eicosanoid signaling) and metabolic pathways (Figure 7). Two gene networks were constructed, related to cell signaling, nucleic acid metabolism and small molecule biochemistry (IPA score = 20), and cellular development, tissue development and function (IPA score = 45), respectively (Figures 8A,B). Accompanying functional annotation clustering analysis revealed the presence of two enriched clusters related to cell to cell signaling (ES = 1.7) and metal-ion binding (ES = 1.3).

## Discussion

Coccidiosis remains one of the costliest diseases for the international poultry industry. Selectively breeding chickens for enhanced resistance to *Eimeria* challenge, and for improved breadth of vaccine response, could provide a tractable strategy to improve coccidiosis control. We conducted two studies using different crosses between the White Leghorn inbred lines 15I and C.B12. Our data confirm that line 15I birds are more susceptible to primary infection with *E. maxima* than line C.B12 by overall PR (Smith et al., 2002; Blake et al., 2006). While the two inbred lines exhibit similar resistance/susceptibility profiles following primary infection with either of the two antigenically distinct *E. maxima* strains, they show radically different levels of protection against heterologous secondary challenge by antigenically distinct strains of the same pathogen (Smith et al., 2002). We therefore investigated the genetic background of resistance to primary and heterologous secondary *E. maxima* W challenges.

The resistance of chickens to *Eimeria* infection has traditionally been quantified using measures such as oocyst output and LS, indicating resistance to PR and parasite-induced pathology, respectively. For the former, the fewer oocysts excreted, the more resistant the chicken. Thus, oocyst shedding is considered to be an indicative trait and an accurate phenotype for calculating resistance to primary infection and subsequent parasite challenges and this method was used in the intercross experiment. However, calculation of oocyst output by fecal flotation and microscopy is labor intensive. Thus, quantitative real-time PCR for parasite genome copies in intestinal tissues was used as an alternative measure of PR in the more recent backcross experiment (Blake et al., 2006). A third trait, serum IL-10, was also quantified for these latter chickens, providing a measure of the innate immune response to *Eimeria* infection (Rothwell et al., 2004; Boulton et al., 2018). IL-10 is produced after *E. maxima* and *E. tenella* primary infection of White Leghorn chickens (lines 15I and C.B12) and *E. tenella* primary infection of commercial broilers (Rothwell et al., 2004; Wu et al., 2016; Boulton et al., 2018). In all these cases, IL-10 was expressed at high levels in infected birds only, and significantly correlated with pathology (lesion scores). Here, GWAS from the backcross experiment identified markers associated with IL-10 that exhibit significant additive genetic variance. These findings, in conjunction with indications that IL-10 is correlated significantly with gross pathology in a commercial population primary infection with *E. tenella* (Boulton et al., 2018), support the use of IL-10 as an accessible early-life biomarker in breeding programs aiming to enhance *Eimeria* resistance to challenge or pathological outcomes.

Although the significance of *E. maxima* in field coccidiosis has been recognized for many years, there has been a limited number of genetic studies investigating host resistance to *E. maxima* primary infection and challenge. A recent study that investigated the genetic background of resistance to high-level *E. maxima* infection using the same HD genotyping array but measuring three different phenotypes (body weight gain, plasma coloration, and β2-globulin in blood plasma) identified several QTL on chromosomes 1, 2, 3, 5, and 10 in commercial Cobb500 broilers (Hamzic et al., 2015). Similar to our findings, Hamzic et al. (2015) found no QTL overlap among their different phenotypes. Interestingly, QTL identified by Hamzic et al. (2015) on chromosome 1 for β2-globulin in blood plasma is nearby (2 Mb difference) QTL found in our study linked to for resistance to heterologous secondary *E. maxima* W challenge. Similar enriched biological pathways related to innate immune responses and metabolic processes were also detected in the two studies with this parasite species.

In other comparable work, Zhu et al. (2003) performed a linkage analysis study investigating chicken resistance in terms of oocyst output following controlled *E. maxima* infection using an F2-intercross between two broiler lines with different susceptibility to primary *E. maxima* infection. Using 119 microsatellite markers one locus associated with *E. maxima* resistance was identified on chromosome 1 (Zhu et al., 2003). Expanding this work, Kim et al. (2006) used nine microsatellite markers located on chromosome 1 to refine this region. According to their results, the peak of QTL was located a considerable genetic distance (i.e., 254 cM) away from the chromosome 1 QTL identified here and in the Hamzic et al. (2015) study. This could be attributed to the use of different chicken lines, *E. maxima* strains, analysis methods, and/or genotyping tools. It is worth mentioning that the power to detect QTL as well as the resolution of their location using a few microsatellites is limited compared to HD genotyping platforms.

Comparison of the re-sequencing data of the two parental chicken lines identified a small number of genes that differ regarding the presence of exonic variants with a putative functional effect on the encoded proteins. Two genes of interest with missense variants located in the candidate regions for resistance to *E. maxima* primary infection encode Phosducin Like 3 (*PDCL3*) and TBC1 Domain Family Member 8 (*TBC1D8*) proteins. These immune-related genes were included in the two networks related to inflammatory response, and cell death and survival, constructed by IPA. PDCL3 acts as a chaperone for the angiogenic vascular endothelial growth factor receptor, controlling its abundance and inhibiting its ubiquitination and degradation, and also modulating activation of caspases during apoptosis (Wilkinson et al., 2004; Srinivasan et al., 2013). *TBC1D8* is involved in the regulation of cell proliferation, calcium ion transportation, and also has GTPase activator activity (Ishibashi et al., 2009).

The genes encoding Thromboxane A Synthase 1 (*TBXAS1*) and Pro-Melanin Concentrating Hormone (*PMCH*) are located in the candidate region and are of interest in resistance to secondary challenge by heterologous *E. maxima* W. *TBXAS1* encodes a member of the cytochrome P450 superfamily of enzymes involved in both immune response and metabolism; it plays a role in drug metabolism, platelet activation and metabolism, and synthesis of cholesterol, steroids, and other lipids (Yokoyama et al., 1991; Miyata et al., 1994). The pro-inflammatory actions of thromboxane receptors have been demonstrated to enhance cellular immune responses in a mouse model (Thomas et al., 2003). *PMCH* encodes a preproprotein that is proteolytically processed to generate multiple protein products, including melanin-concentrating hormone (MCH) that stimulates hunger and may additionally regulate energy homeostasis, reproductive function, and sleep (Viale et al., 1997; Chagnon et al., 2007). In a further mouse model, MCH has also been reported as a mediator of intestinal inflammation (Kokkotou et al., 2008). Although, the genes mentioned above are good functional candidates for resistance to primary infection and heterologous challenge with *E. maxima*, further studies are needed to confirm the present results and identify the actual causative genes and mutations.

The immune interactions between an intracellular pathogen and a host are complex and vary as a consequence of the survival mechanisms that have evolved in both (Blake et al., 2011; Blake and Tomley, 2014). It has been suggested that host control of challenge with *Eimeria*, an obligate intracellular pathogen, requires a strong inflammatory, mostly cell mediated response (Shirley et al., 2005; Dalloul and Lillehoj, 2006). Also, host innate immune responses have been detected during initial pathogen exposure in several studies (Kim et al., 2008; Pinard-van der Laan et al., 2009; Wu et al., 2016; Boulton et al., 2018). According to our findings, several gene networks and pathways relating to innate, humoral and cell-mediated, immune responses were highlighted from the gene products located in the candidate regions for resistance to primary *Eimeria* infection. Among the canonical pathways, IL-10 signaling was the most significant, with relevance as a regulator of cytokines such as interferon- (IFN-) γ. These findings agree with previous studies of *Eimeria* resistance that have highlighted IFN γ and tumor necrosis factor (TNF) nodes as crucial (Pinard-Van Der Laan et al., 1998; Smith and Hayday, 2000a,b; Bacciu et al., 2014), since IL-10 downregulates IFNγ production (Schaefer et al., 2009).

## Conclusion

We identified genomic regions, putative candidate genes, canonical pathways and networks involved in the underlying molecular mechanisms of chicken resistance to *E. maxima* primary infection and to secondary heterologous *E. maxima* strain challenge. More emphasis should be placed on the relevant mechanisms for disease resistance, the response to secondary heterologous strain challenge and the role of IL-10 induction in immune responses to intestinal challenge in the future selective breeding of chickens.

## Availability of Supporting Data

The resequencing data used in this study is available in NCBI dbSNP at the following web page: http://www.ncbi.nlm.nih.gov/SNP/snp_viewBatch.cgi?sbid=1062063.

## Author Contributions

AS, PK, SB, FT, and DB devised the overall strategy and obtained funding. PK, SB, FT, and DB conceived the backcross experiments. PMH and KB devised the backcross breeding. MN managed the backcross trials and performed qPCR and DNA extraction assisted by KH and KB. Backcross phenotype collection was carried out by MN, KH and KB, while DB scored lesions. ZW performed IL-10 assays assisted by KB. KB prepared backcross DNA for genotyping and carried out all backcross analyses with input from AP, VR, and OM. AS designed the intercross trials with input from NB and these were carried out by PH and AA. AP performed an initial analysis of the intercross data with input from OM and KB. Pathway and resequencing analyses were performed by AP and KB. The manuscript was drafted by KB and AP with input from all other authors except PMH, SB, NB, and PK. AS, DB, FT, DH, and AP assisted in the interpretation of results.

## Conflict of Interest Statement

The authors declare that the research was conducted in the absence of any commercial or financial relationships that could be construed as a potential conflict of interest. The handling Editor declared a shared affiliation, though no other collaboration, with several of the authors, NB, PH, AA, and AS at time of review.

## Footnotes

1 http://www.ensembl.org/Tools/VEP

2 https://www.ncbi.nlm.nih.gov/assembly/GCF_000002315.4/

3 http://www.ensembl.org/biomart/martview/

4 www.ingenuity.com
